# Glucocorticoid-induced leucine zipper as a context-dependent central integrator of innate and adaptive immune homeostasis

**DOI:** 10.3389/fimmu.2026.1947003

**Published:** 2026-09-22

**Authors:** Wassim Y. Almawi, Rita Nemr

**Affiliations:** 1 Faculty of Sciences, El-Manar University, Tunis, Tunisia; 2 Department of Internal Medicine, LAU Medical Center - Rizk Hospital, Beirut, Lebanon

**Keywords:** adaptive immunity, GILZ, glucocorticoids, immunometabolism, inflammation, innate immunity

## Abstract

Glucocorticoid-induced leucine zipper (GILZ) was originally identified as an immediate transcriptional target of glucocorticoid receptor (GR) activation and viewed primarily as a mediator of the anti-inflammatory and immunosuppressive effects of glucocorticoids (GCs). Over the last two decades, this perspective has evolved considerably, and GILZ is now recognized as a widely expressed regulatory protein in most immune cells and many non-immune tissues. In addition to GCs, anti-inflammatory cytokines, including interleukin-10 (IL-10) and transforming growth factor-β (TGF-β), along with metabolic and tissue-derived signals, control GILZ expression. These diverse inputs suggest that GILZ acts within broad physiological networks that extend beyond traditional inflammatory pathways. GILZ’s biological activities include regulating cell differentiation, survival, metabolism, tissue repair, and immune responses. Rather than acting through intrinsic enzymatic activity, GILZ exerts its effects mainly through selective interactions with transcription factors and signaling molecules. Through these interactions, it modulates how cells integrate and interpret multiple environmental and intracellular cues, thereby influencing cellular behavior in a context-dependent manner. This review integrates established and emerging evidence into a context-dependent framework for GILZ function across immune and non-immune systems. Rather than defining new functions, it examines how GILZ interactions produce distinct outcomes depending on cellular identity, tissue environment, activation state, and inflammatory timing. Emphasis is placed on immune-cell plasticity, immunometabolic adaptation, tissue homeostasis, inflammation resolution, and their implications for the therapeutic potential and limitations of GILZ modulation. We propose that the biological significance of GILZ is best understood, not as that of a master suppressor of immunity, but as a context-dependent integrator that helps determine how cells transition between inflammatory, regulatory, reparative, and homeostatic states.

## 1 Introduction

Encoded by the TSC22D3 gene, the glucocorticoid-induced leucine zipper (GILZ) was originally identified in studies of glucocorticoid (GC)-responsive genes in thymocytes following glucocorticoid receptor (GR) activation (1). GILZ was initially characterized for its capacity to inhibit nuclear factor-κB (NF-κB) and activator protein-1 (AP-1) transcriptional activity, providing a molecular explanation for key anti-inflammatory properties attributed to therapeutic glucocorticoids (GCs) (1, 2). Consistent with its rapid induction kinetics and its ability to attenuate inflammatory gene transcription in multiple cell types, early functional characterization identified GILZ as a downstream mediator of GC-induced immunosuppression (3, 4). While this labelling was biologically correct, it was ultimately found to be incomplete. Subsequent investigations showed that GILZ expression is regulated not only by GCs but also by a myriad of physiological stimuli, including IL-10 and TGF-β (5), pathogen-associated molecular patterns (6), metabolic signals, and tissue-specific stromal factors (5, 7).

GILZ is expressed in virtually all major immune-cell populations, including macrophages, dendritic cells (DCs), neutrophils, innate lymphoid cells, T lymphocytes, B lymphocytes, and regulatory T cells (6, 8), as well as in numerous non-immune populations, including epithelial cells, endothelial cells, fibroblasts, adipocytes, hepatocytes, and neural cells. The documented biological activities of GILZ span inflammatory regulation, cellular differentiation, apoptotic signaling (6, 9), metabolic reprogramming, and tissue repair (4, 10), a functional breadth inconsistent with a protein viewed solely as a glucocorticoid effector.

The molecular basis for the functional diversity of GILZ lies in its structural organization (2). Unlike kinases, phosphatases, or transcription factors that initiate signaling cascades independently, GILZ acts primarily through selective protein--protein interactions (6, 7). It lacks intrinsic enzymatic activity and instead modulates the assembly, stability, localization, or transcriptional competence of regulatory protein complexes within existing signaling networks (6, 11). Consequently, its biological output reflects not the molecule itself but the regulatory environment in which those interactions occur (7). Accordingly, GILZ has been described as a molecular integrator, a protein whose functional significance derives from its ability to modify how immune cells translate physiological signals into adaptive responses (12), rather than directing those responses from a position of hierarchical control (8, 13).

This framework explains why studies of GILZ often yield seemingly contradictory results. For example, GILZ-mediated inflammatory suppression was reported in one setting (13, 14), whereas promotion of differentiation or survival was observed in a different model (15), and stabilization of regulatory T-cell (Treg) and tolerogenic antigen-presenting cell (APC) programming was also reported (7). However, these findings need not be interpreted as evidence of unrelated or contradictory biological functions, since they reflect the context-dependent nature of the GILZ protein, which adjusts the weighting of competing signaling inputs according to cellular identity, activation state, endocrine environment, and tissue context (11). Recognizing this is essential not only for biological understanding but also for evaluating the translational potential of GILZ-targeted interventions.

This review advances a context-dependent framework for understanding GILZ, positioning it not as a regulator with fixed functions but as an integrator of inflammatory, endocrine, metabolic, differentiation, survival, and tissue-derived signals whose effects depend on cellular and biological context. By unifying immune-cell plasticity, immunometabolism, tissue homeostasis, and inflammation resolution, this framework helps explain how established signaling networks collectively shape inflammatory, regulatory, reparative, and homeostatic phenotypes. We examine the structural and molecular basis of this regulatory versatility, including GILZ architecture, isoform diversity, expression, and signaling interactions, and evaluate its roles in innate and adaptive immunity, macrophage state transitions, regulatory antigen-presenting cells, and T-cell differentiation. We also discuss implications for immunometabolism, tissue homeostasis, and therapeutic targeting, while explicitly distinguishing experimentally demonstrated mechanisms from functions that remain inferential or context-dependent.

## 2 Molecular organization, structure, and regulatory mechanisms

### 2.1 Molecular architecture and isoform diversity of GILZ

GILZ is a member of the TSC-22 (TGFβ-stimulated clone 22, also termed TFE) family, a group of proteins defined by a conserved TSC-22 domain and a C-terminal leucine zipper (2, 16). The TSC-22 domain mediates homo- and heterodimerization and is critical for the protein-protein interactions that underlie GILZ function (1). Alternative transcripts from TSC22D3 have been described, generating variants historically termed long (L-GILZ), mid (M-GILZ), and short (S-GILZ), which differ mainly in their N-terminal regions (11, 16, 17) (**Figure 1**). However, isoform existence, expression, and function are distinct claims; distinct transcripts or predicted proteins alone do not demonstrate endogenous expression or function.

**Figure 1:**
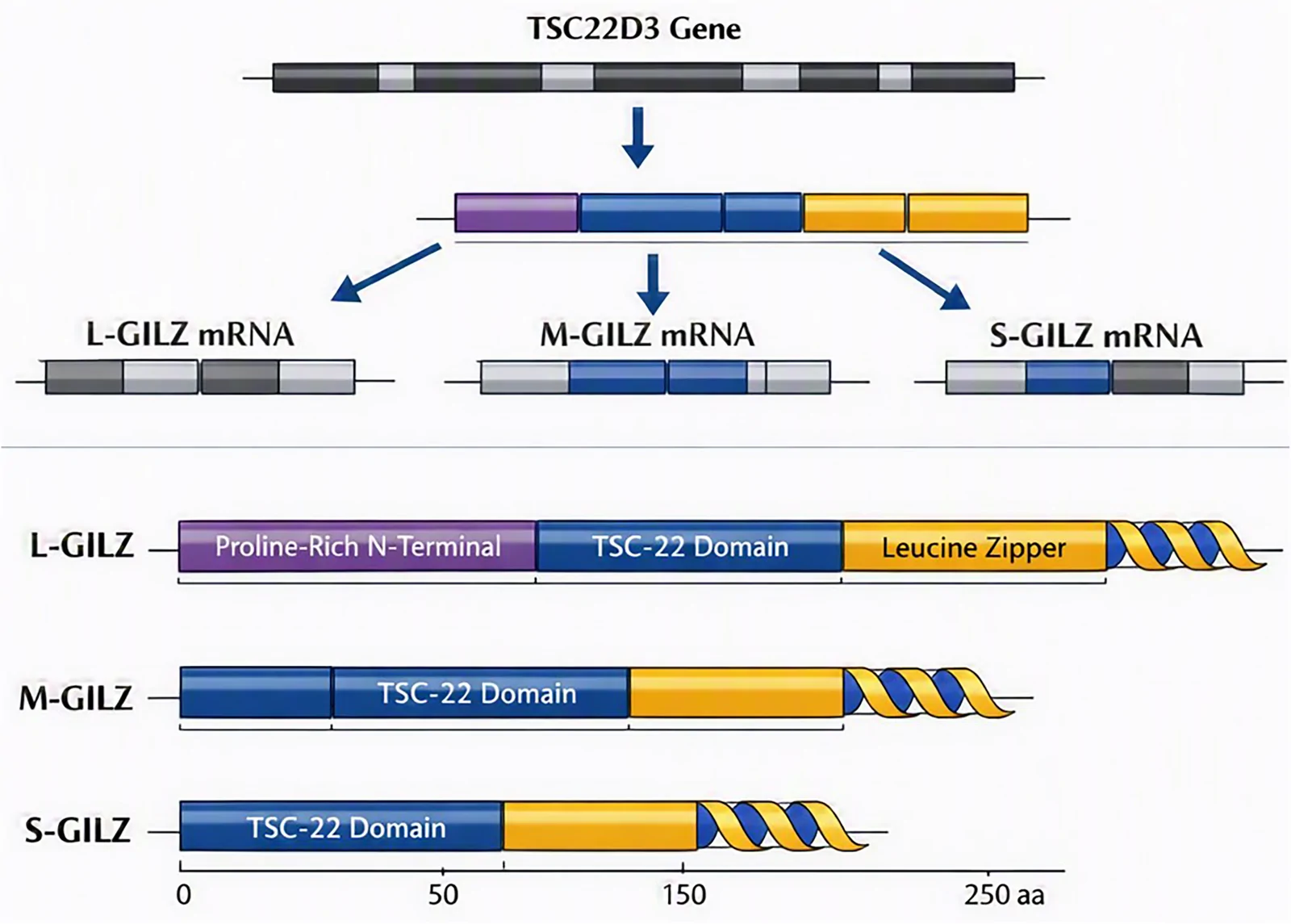
Reported transcript and protein isoforms derived from the *TSC22D3* locus. Schematic representation of the *TSC22D3* gene and its principal transcript variants encoding long (L-GILZ), mid (M-GILZ), and short (S-GILZ) GILZ isoforms. All isoforms retain the conserved TSC-22 domain and C-terminal leucine zipper, whereas N-terminal extensions differ, most prominently in L-GILZ. Created with BioRender.com and Adobe Illustrator.

Identified as a distinct GILZ variant by Bruscoli and colleagues (16), L-GILZ contains an extended proline-rich N-terminal region absent from the shorter isoforms. Functional studies in specific reproductive and lymphocyte-related experimental models demonstrated that L-GILZ interacts with additional regulatory proteins, including Ras-family signaling pathways, and is highly expressed in spermatogonia and primary spermatocytes, where it plays an essential role in spermatogenesis (17). Genetic deletion of the *Tsc22d3/Gilz* locus results in major defects in germ-cell development, impaired spermatogenesis, and male infertility, providing some of the strongest *in vivo* evidence currently available for isoform-specific GILZ function.

Although additional transcript and protein variants, including M-GILZ and S-GILZ, have also been reported, evidence for their endogenous expression patterns and specialized biological functions is more limited. Whereas available studies suggest that the regulatory output of the *TSC22D3* locus extends beyond a single protein product, the physiological significance of several reported isoforms remains to be fully established. S-GILZ has been extensively investigated in inflammatory and hematopoietic models and is one of the better-characterized GILZ forms in these contexts (16). However, observations supporting context-specific functions of L-GILZ and S-GILZ should not be extrapolated to all *TSC22D3* products (16, 17), as evidence supporting M-GILZ as a distinct endogenous protein with defined tissue distribution and reproducible isoform-specific functions remains comparatively limited. Consequently, M-GILZ is best regarded as a reported *TSC22D3* variant rather than a functionally equivalent counterpart to the better-characterized isoforms.

Because many studies assess “GILZ” or manipulate *TSC22D3* without demonstrating isoform specificity, biological effects should generally be attributed to GILZ/TSC22D3 unless direct evidence identifies the responsible isoform. Thus, the relative contributions of individual GILZ isoforms remain incompletely defined and warrant further investigation. Future studies combining transcript-resolved sequencing, isoform-selective proteomics, endogenous tagging, spatial profiling, and isoform-specific genetic perturbation will be required to determine whether reported differences among GILZ variants reflect genuine *in vivo* specialization or context-dependent observations from selected experimental systems.

### 2.2 Signaling networks and molecular targets of GILZ

The molecular interactions through which GILZ regulates cellular behavior are well established and have been extensively reviewed elsewhere (**Figure 2**). Interaction between GILZ and the NF-κB p65 subunit reduces nuclear translocation and transcriptional activity at the promoters of inflammatory gene promoters (2), leading to suppression of canonical proinflammatory cytokines (18, 19) (**Figure 2**). Reviews summarizing these interactions have been published elsewhere (2, 6). Parallel interactions with AP-1 complex (c-Jun, c-Fos) inhibit stress-induced and cytokine-driven gene transcription, without entirely blocking AP-1 pathway engagement, thereby restraining stress-induced and cytokine-driven gene expression (20). Review discussions are provided elsewhere (5). GILZ overexpression in the absence of GCs recapitulates many of the anti-inflammatory effects attributed to GR transactivation (20), establishing GILZ as an autonomous inflammatory modulator rather than a simple GC effector molecule (7, 21).

**Figure 2:**
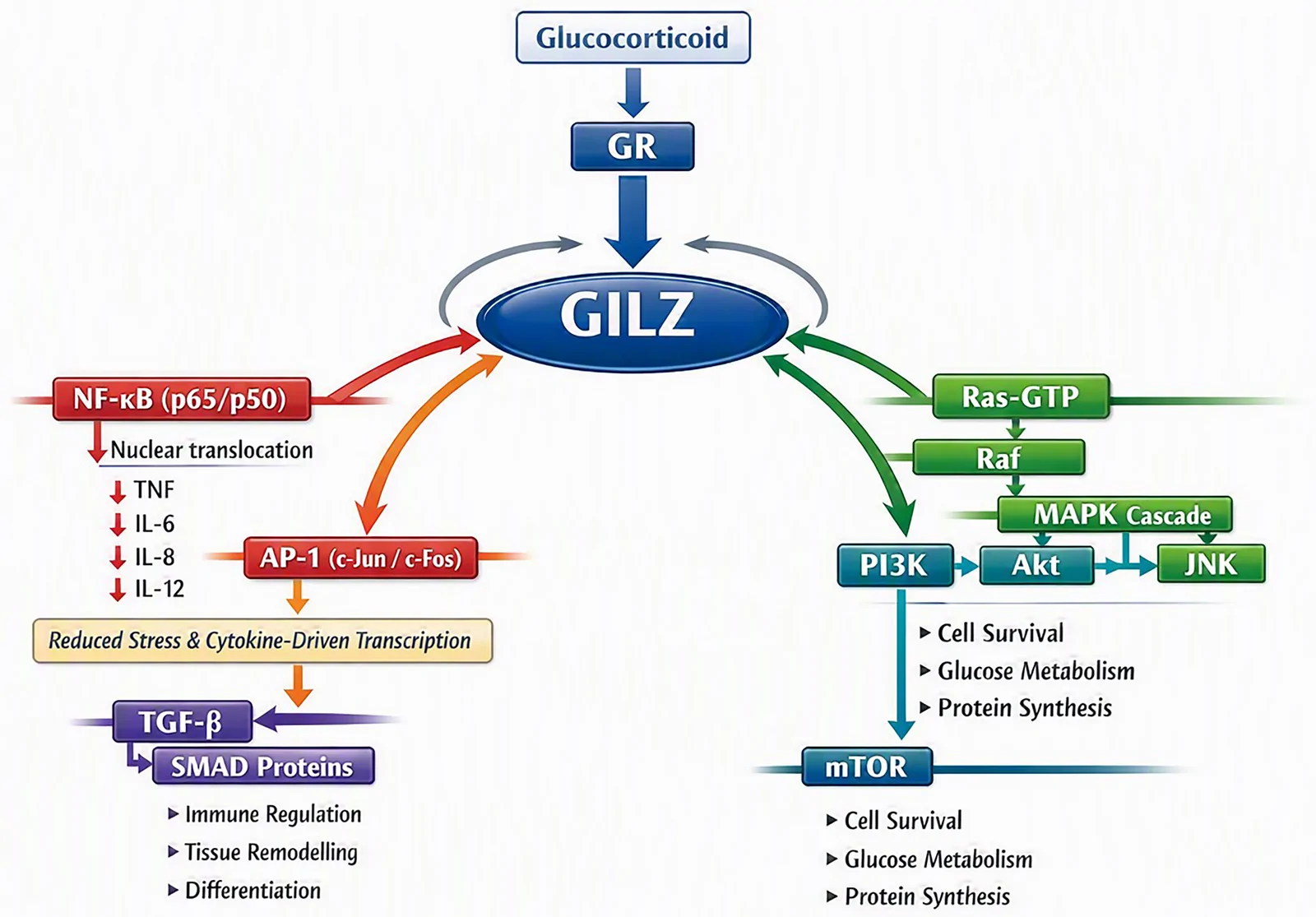
Context-dependent integration of inflammatory, metabolic, differentiation, and survival signaling by GILZ. By interacting with NF-κB (p65/p50) and AP-1 (c-Jun/c-Fos), GILZ suppresses pro-inflammatory gene transcription. Additional interactions with Ras–MAPK, PI3K–Akt–mTOR, SMAD, and glucocorticoid receptor pathways position GILZ as a context-dependent regulator of cellular activation, survival, and homeostasis. Rather than acting as a linear signaling molecule, GILZ is proposed to function as a context-dependent regulator whose biological effects emerge from interactions among multiple interconnected signaling pathways. Created with BioRender.com and Adobe Illustrator.

GILZ also interacts with Ras--MAPK axis components, including Raf kinase and Ras GTPases, attenuating the activation of the MAP kinase cascade (17, 18), leading to reduced downstream ERK and JNK signaling involved in regulating the proliferation, differentiation, and stress responses (17). GILZ suppresses PI3K-Akt signaling, including mTORC2-dependent Akt phosphorylation, thereby attenuating downstream pro-survival, metabolic, and growth-promoting signaling (22). In addition, by inhibiting the PI3K-Akt pathway and relieving Akt-mediated suppression of FOXO3a, GILZ enhances FOXO-dependent apoptosis and promotes FOXO-mediated programs involved in antioxidant defense and cell-cycle control (6, 22). Furthermore, through its interactions with SMAD proteins, GILZ is positioned at the TGF-β superfamily signaling interface. Its direct interaction with the GR also suggests a role in autoregulatory feedback circuits that can either enhance or limit GILZ induction, depending on the stimulus intensity and cellular context (7, 23).

This interaction network appears less dependent on any individual pathway than on the mechanism by which GILZ integrates information across pathways. First, the pathways engaged by GILZ are extensively cross-connected and continuously exchange information that governs immune-cell phenotype (1, 6, 7). By intersecting multiple nodes within this network simultaneously, GILZ influences how competing physiological signals are balanced rather than blocking any single pathway independently (1, 5). Second, none of GILZ’s documented protein interactions requires enzymatic activity or post-translational modification of GILZ itself; the protein operates through binding affinity and structural constraints on interaction partners (12, 24). This suggests that GILZ levels can be rapidly adjusted to alter network function without changes to complex enzymatic machinery, a property consistent with its role as a rapid-response mediator (12, 25). Third, the consequences of GILZ-mediated interactions vary substantially among cell types because the stoichiometry, isoform expression, and subcellular localization of interaction partners differ according to cellular identity and activation history (6, 7). In turn, this generates distinct biological outcomes from the same fundamental molecular interaction.

### 2.3 Glucocorticoid-independent regulation of GILZ

The GC-independent regulation of GILZ is key for understanding the broader regulatory context in which GILZ operates. In this regard, the *TSC22D3* promoter contains binding sites not only for glucocorticoid response elements (GREs), but also for STAT6, STAT3, Smad3/4, and additional transcription factors activated by cytokines and growth factors independent of GC signaling (5, 26, 27). IL-10 induces GILZ through STAT3-dependent mechanisms in macrophages and lymphocytes (8); TGF-β induces it through Smad3 in both immune and non-immune cells (7, 28); IL-4 drives GILZ expression through STAT6 in macrophages and mast cells (29). These pathways collectively establish GILZ as a convergence point for anti-inflammatory cytokine signaling that operates in parallel with, and is not dependent upon, GR activation (23, 30). This regulatory scheme indicates that GILZ expression is determined by the integrated output of multiple anti-inflammatory signals, any one of which may be sufficient to induce it (23, 30). This positions GILZ as a molecular regulator of the anti-inflammatory cytokine tone within specific tissue microenvironments, more than a reporter of systemic GC status.

### 2.4 Multilayered regulation of GILZ expression and function

GILZ expression is regulated at the transcriptional and post-transcriptional levels and involves multiple molecular mechanisms that control its expression, stability, and functional activity. GILZ expression is subject to microRNA-mediated suppression, as evidenced by the ability of miR-98 and let-7 family members to target the TSC22D3 3′-UTR in macrophages and T cells under proinflammatory conditions (6, 29), providing a mechanism by which inflammatory cytokines attenuate GILZ-mediated regulatory tone (29, 31). GILZ protein stability is governed by ubiquitin-proteasomal degradation (32), and emerging evidence suggests that GILZ function is post-translationally regulated, including by SUMO-associated modification and proteasome-dependent turnover, which in turn influence its downstream biological effects (32, 33). Epigenetic regulatory mechanisms, such as GRE occupancy, histone acetylation at the *TSC22D3* locus, and CpG methylation, further influence cell-type and activation-state-specific expression patterns (20, 34, 35). Taken together, these results highlight the importance of regulating GILZ availability and identify potential therapeutic strategies that do not rely on GR activation.

## 3 Regulation of innate immunity by GILZ

GILZ is a key regulator of innate immunity and an important mediator of GC-induced anti-inflammatory changes (6, 28). GILZ is expressed in macrophages, dendritic cells, neutrophils, and other innate immune cells, and it suppresses inflammatory responses (29, 30, 36), largely by modulating pathways that drive pro-inflammatory gene expression. Through its interactions with NF-κB, AP-1, and other transcription factors, GILZ maintains immune homeostasis and promotes the resolution of inflammation.

### 3.1 Macrophages

Macrophages are among the best-characterized innate immune cell populations in which GILZ exerts its immunoregulatory functions and provide an informative model for the context-dependent interpretation of GILZ biology (8, 28, 29). Rather than assigning GILZ to a fixed anti-inflammatory phenotype, the available evidence suggests that its effects are better understood in relation to the dynamic sequence of macrophage activation, adaptation, resolution, and tissue repair. In this setting, GILZ integrates inflammatory signals with endocrine, metabolic, and tissue-derived cues, since its expression is sensitive to environmental factors and is induced by anti-inflammatory cytokines, including IL-10 and IL-4, as well as by GCs (6, 30). Exposure to pro-inflammatory factors such as lipopolysaccharide (LPS), TNFα, and interferon-γ (IFN-γ) suppresses GILZ expression (8, 30, 37). Owing to this reciprocal regulation, GILZ constitutes a key molecular integrator of endocrine and inflammatory signals, linking alterations in the tissue microenvironment to macrophage functional behavior (6, 8, 30).

Across experimental models, GILZ has emerged as a key negative regulator of macrophage activation. Reduced GILZ expression is linked to increased NF-κB activity (37), heightened production of pro-inflammatory mediators (4, 30), greater responsiveness to TLR stimulation (38), and elevated ROS generation (39, 40). In contrast, increased GILZ expression limits these inflammatory responses while preserving antimicrobial functions (4, 8). These findings suggest that GILZ primarily sets the threshold for macrophage activation rather than broadly suppressing macrophage function. This indicates that the regulatory role of GILZ may help maintain effective host defenses while preventing excessive inflammation and tissue damage (31, 41).

#### 3.1.1 GILZ and macrophage phenotypic plasticity

Rather than fitting into the traditional M1/M2 polarization model, macrophages are increasingly viewed as highly plastic cells that adapt their phenotype and function in response to changes in the microenvironment (42). Within this context, GILZ is regarded as a regulator of macrophage phenotypic adaptation, more than a marker of a specific macrophage phenotype (43). GILZ modulates macrophage activation programs across different tissue environments by integrating anti-inflammatory and stress-related signals with inflammatory pathways (4, 41). Emerging evidence indicates that GILZ influences macrophage responses during the initiation and resolution of inflammation, while also supporting processes involved in tissue repair and the restoration of homeostasis (41, 43). Collectively, this supports a role for GILZ as an important molecular checkpoint that facilitates macrophage plasticity in changing microenvironments.

#### 3.1.2 Promotion of efferocytosis and resolution

zEfferocytosis is a critical mechanism for resolving inflammation and is linked to the stimulation of anti-inflammatory and pro-resolving mediators, including IL-10, TGF-β, and specialized pro-resolving lipid mediators (43, 44). GILZ plays a key role in promoting macrophage efferocytosis by enhancing the expression of phosphatidylserine-binding receptors and efferocytic mediators (including MerTK and Tim-4) (45), thereby facilitating apoptotic cell uptake (41, 43). In addition, GILZ limits inflammatory signaling associated with non-efferocytic phagocytic pathways (6). Owing to its effects on efferocytosis, these findings assign a role to GILZ in promoting a self-reinforcing resolution program that facilitates the transition of macrophages from inflammatory to reparative phenotypes.

#### 3.1.3 Regulation of reparative macrophage functions

In addition to its role in efferocytosis, GILZ promotes macrophage reprogramming during the resolution phase of inflammation (41). GILZ reportedly regulates gene expression programs linked to tissue repair and the restoration of homeostasis by modulating signaling pathways involving TGF-β-SMAD and FOXO transcription factors (9, 46). GILZ-expressing macrophages display enhanced pro-resolving and reparative functions, including increased phagocytosis, enhanced IL-10 signaling, and extracellular matrix remodeling, which in turn support tissue repair and the restoration of hemostasis (8, 47). Consistent with a role in tissue repair and remodeling, GILZ overexpression is associated with increased matrix metalloproteinase activity and altered expression of genes involved in extracellular structure organization (8). Although the underlying mechanisms remain incompletely understood, current evidence implicates GILZ in regulating pro-resolving macrophage responses and tissue-remodeling programs associated with the restoration of homeostasis (43).

#### 3.1.4 Tissue-resident macrophages and homeostasis

GILZ expression is not restricted to inflammatory conditions, and both constitutive and inducible forms have been reported in several tissue-resident macrophage populations, including alveolar macrophages, Kupffer cells, intestinal macrophages, and microglia (30, 48). These resident macrophages perform essential housekeeping functions that support tissue integrity and immune homeostasis under steady-state conditions (49). Although the role of GILZ in tissue-resident macrophages remains incompletely defined, its constitutive expression in tissue macrophages and its established inhibitory effects on macrophage activation support a role in maintaining macrophage homeostasis and limiting excessive inflammatory responses (30). This suggests that GILZ contributes to macrophage homeostasis, although its tissue-specific functions and underlying mechanisms remain incompletely characterized.

#### 3.1.5 GILZ as a regulator of macrophage immunometabolism

Macrophage activation is closely linked to cellular metabolism, and emerging evidence suggests that GILZ helps coordinate metabolic and inflammatory signaling pathways (8, 50). GILZ interacts with regulatory networks that include PI3K-Akt-mTOR and FOXO-dependent signaling (51) and influences mitochondrial function and gene programs involved in extracellular structure organization (13, 52). Supporting this view, findings show that GILZ overexpression alters mitochondrial activity and reduces mitochondrial respiration (39), and that GILZ deficiency is associated with increased reactive oxygen species production and enhanced inflammatory responses (40, 50). Together, these findings indicate that GILZ expression is associated with metabolic and mitochondrial changes linked to macrophage activation and functional specialization (4, 8); however, whether GILZ directly drives metabolic reprogramming remains unresolved.

#### 3.1.6 Integrative role of GILZ in macrophage plasticity

Collectively, current evidence identifies GILZ as a key regulator of macrophage plasticity (6). Rather than defining a discrete activation state, GILZ modulates multiple processes that shape macrophage function, including inflammatory signaling, phagocytosis, efferocytosis, resolution-associated responses, and pathways linked to tissue remodeling (41, 43). Accordingly, available evidence suggests that GILZ contributes to transitions among inflammatory, resolving, and homeostatic states, although the relative contribution of direct GILZ-dependent mechanisms versus secondary effects of broader regulatory networks remains incompletely defined (**Figure 3**). This integrative role may underlie the broad influence of GILZ on macrophage function in both physiological and pathological settings.

**Figure 3:**
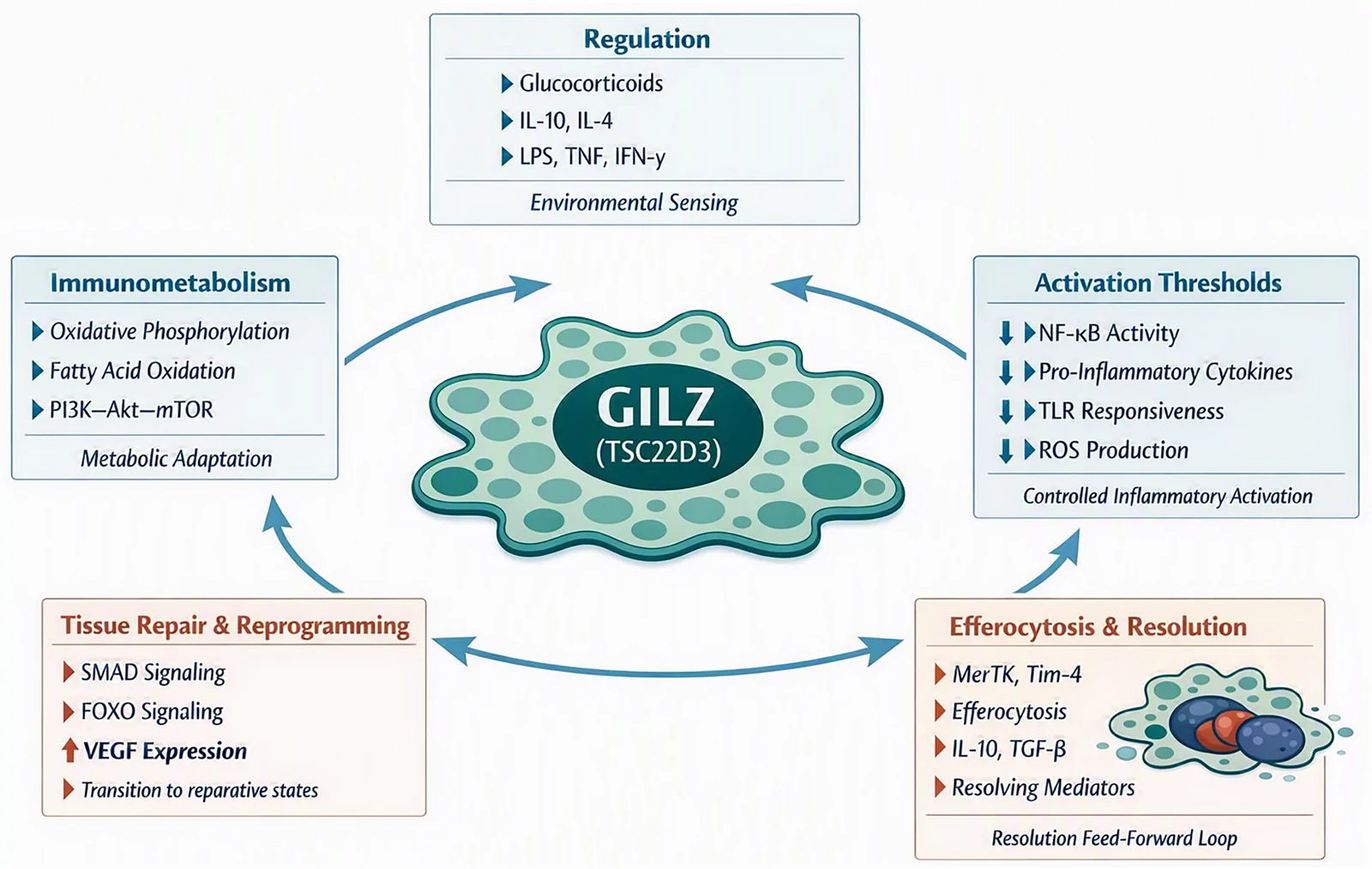
Regulation of macrophage phenotypic transitions across inflammation, resolution, tissue repair, and homeostasis by GILZ. Regulated by glucocorticoids, IL-10, IL-4, LPS, TNF, and IFN-γ, GILZ coordinates four interconnected pathways: suppression of NF-κB, pro-inflammatory cytokine production, TLR signaling, and ROS generation; metabolic reprogramming through oxidative phosphorylation, fatty acid oxidation, and PI3K–Akt–mTOR; efferocytosis and resolution via MerTK/Tim-4, IL-10, and TGF-β; and tissue repair through SMAD/FOXO signaling and VEGF induction. Crosstalk among these pathways positions GILZ at the intersection of inflammatory, metabolic, reparative, and homeostatic responses, highlighting a context-dependent role in shaping macrophage behavior rather than controlling any single pathway or activation state. Created with BioRender.com and Adobe Illustrator.

### 3.2 Dendritic cells

GILZ is constitutively expressed in dendritic cells (DCs) and further induced by glucocorticoids, IL-10, and TGF-β (53, 54). Increased GILZ expression in human and murine DCs is associated with a regulatory phenotype marked by reduced expression of the costimulatory molecules CD80 and CD86 and of CD40 (a TNF receptor superfamily member involved in DC--T cell cognate interactions) (55), decreased IL-12 production, enhanced IL-10 secretion, and an increased capacity to promote regulatory T-cell differentiation while limiting Th1- and Th17-cell responses (16, 54). Conversely, genetic deletion or experimental reduction of GILZ expression enhances DC immunogenicity, impairs tolerogenic function, and compromises mechanisms that contribute to peripheral tolerance (6, 56).

GILZ regulates the mechanisms that enable DCs to interpret and integrate environmental signals before initiating adaptive immune responses (57). Rather than constitutively inhibiting DC activation, GILZ balances inflammatory and regulatory responses, shaping antigen presentation and T-cell priming (36, 57). Sustained GILZ expression under homeostatic conditions supports tolerance to self-antigens and non-pathogenic environmental antigens (36, 58). In contrast, inflammatory stimuli associated with infection or tissue damage are accompanied by reduced GILZ expression (6), facilitating DC maturation and the development of effective immune responses (54, 58). Restoration of GILZ expression during the resolution phase is thought to help re-establish immune homeostasis (59).

Emerging evidence suggests that GILZ contributes to cellular processes that underpin the metabolic control of dendritic-cell function, including antigen uptake, processing and tolerogenic programming (54, 58). Elevated GILZ expression promotes tolerogenic dendritic-cell functions, including regulatory T-cell expansion and suppression of antigen-specific T-cell responses. These effects align with IDO-dependent immune regulation (36, 57), although a direct link between GILZ expression and IDO activity in dendritic cells remains incompletely defined (60). This suggests that GILZ may couple metabolic adaptations to tolerogenic DC function through its association with IDO-dependent immunoregulatory pathways (55), potentially extending its role beyond transcriptional control of inflammatory signaling to regulating the local immune microenvironment (61). This interaction is particularly relevant in settings such as transplantation and autoimmune disease, where glucocorticoid-induced tolerance has been linked to IDO-dependent mechanisms.

### 3.3 Neutrophils

Neutrophils undergo rapid constitutive apoptosis after entering tissues under homeostatic conditions, promoting efferocytic clearance and preventing persistent inflammation (62, 63). Glucocorticoids delay neutrophil apoptosis by activating PI3K--Akt-dependent survival pathways and modulating Bcl-2 family proteins, including suppression of pro-apoptotic Bim (33, 64). Although GILZ mediates several anti-inflammatory actions of GCs in neutrophils, its role in neutrophil survival appears distinct, as GILZ expression has been associated with promoting rather than inhibiting apoptosis (13). Although GCs prolong neutrophil survival, increasing evidence indicates that GILZ contributes to the subsequent resolution phase by promoting neutrophil apoptosis and limiting persistent inflammation (33). This raises the possibility that GILZ helps fine-tune neutrophil lifespan during inflammatory responses (28, 33).

GILZ modulates neutrophil trafficking by regulating the CXCR2-CXCR4 chemokine receptor axis, which balances neutrophil mobilization to inflamed tissues with retention in the bone marrow (65). Experimental evidence indicates that GILZ regulates neutrophil accumulation at inflammatory sites, thereby contributing to the control of inflammatory responses (66). Emerging evidence suggests that GILZ regulates NET formation. Given that excessive NET release has been implicated in tissue damage, thrombosis, and the propagation of autoimmune inflammation, GILZ-mediated control of NETosis may help limit the pathological consequences of neutrophil activation (67). Collectively, these findings identify GILZ as a regulator of neutrophil responses that fine-tunes the magnitude, localization, and resolution of acute inflammation, rather than acting solely as a determinant of neutrophil survival or antimicrobial activity (33, 66).

### 3.4 Innate lymphoid cells

Compared with its well-characterized functions in myeloid cells, its role in innate lymphoid cell (ILC) biology remains largely unexplored. Functional insights from other immune-cell populations may help generate testable hypotheses for future investigations. Several pathways central to ILC development, maintenance, and effector function, including GC signaling, PI3K--Akt-dependent responses, cellular metabolism, and transcriptional regulation, are known GILZ targets in other immune-cell populations (18, 68). ILC2s contribute to type 2 immunity, tissue repair, and metabolic homeostasis and are subject to GC-mediated regulation. Given that GILZ is a major downstream mediator of GC signaling in several immune-cell populations (69), its potential role in ILC2 biology warrants investigation. Similarly, ILC3s are essential for maintaining mucosal barrier integrity and host--microbiota homeostasis. Cytokine-dependent pathways regulate their development and effector functions, and GILZ modulates these pathways in other immune-cell populations, including IL-10- and TGF-β-associated networks (23, 69). Consequently, defining GILZ’s role in ILC subsets is important for understanding barrier tissue homeostasis and immune adaptation, but current hypotheses largely extrapolate from other immune cells because direct evidence in ILCs remains limited.

## 4 GILZ in adaptive immune regulation

Adaptive immune responses depend on the coordinated activation, differentiation, and regulation of antigen-specific lymphocytes (70). As a key downstream effector of GC signaling, GILZ helps maintain adaptive immune homeostasis by modulating T- and B-cell responses, limiting excessive immune activation, and promoting regulatory pathways (21, 53, 71). Through these actions, GILZ plays a key role in controlling inflammation, maintaining immune tolerance, and shaping adaptive immune responses.

### 4.1 GILZ and regulatory T cells

Among adaptive immune cell populations, the interaction between GILZ and regulatory T (Treg) cells is the best characterized. GCs, TGF-β, and IL-2 induce GILZ expression, which helps maintain the Treg phenotype (**Figure 4**) (7, 23). Experimental evidence indicates that GILZ promotes FoxP3 expression and supports Treg differentiation and function (23). By contrast, GILZ deficiency is associated with reduced Treg frequency, impaired suppressive capacity, and increased susceptibility to inflammatory and autoimmune disease (23, 31). Conversely, increased GILZ expression reportedly strengthens the regulatory properties of T cells and favors the development of anti-inflammatory immune responses (16). These findings identify GILZ as a key component of the molecular network that sustains peripheral immune tolerance.

**Figure 4:**
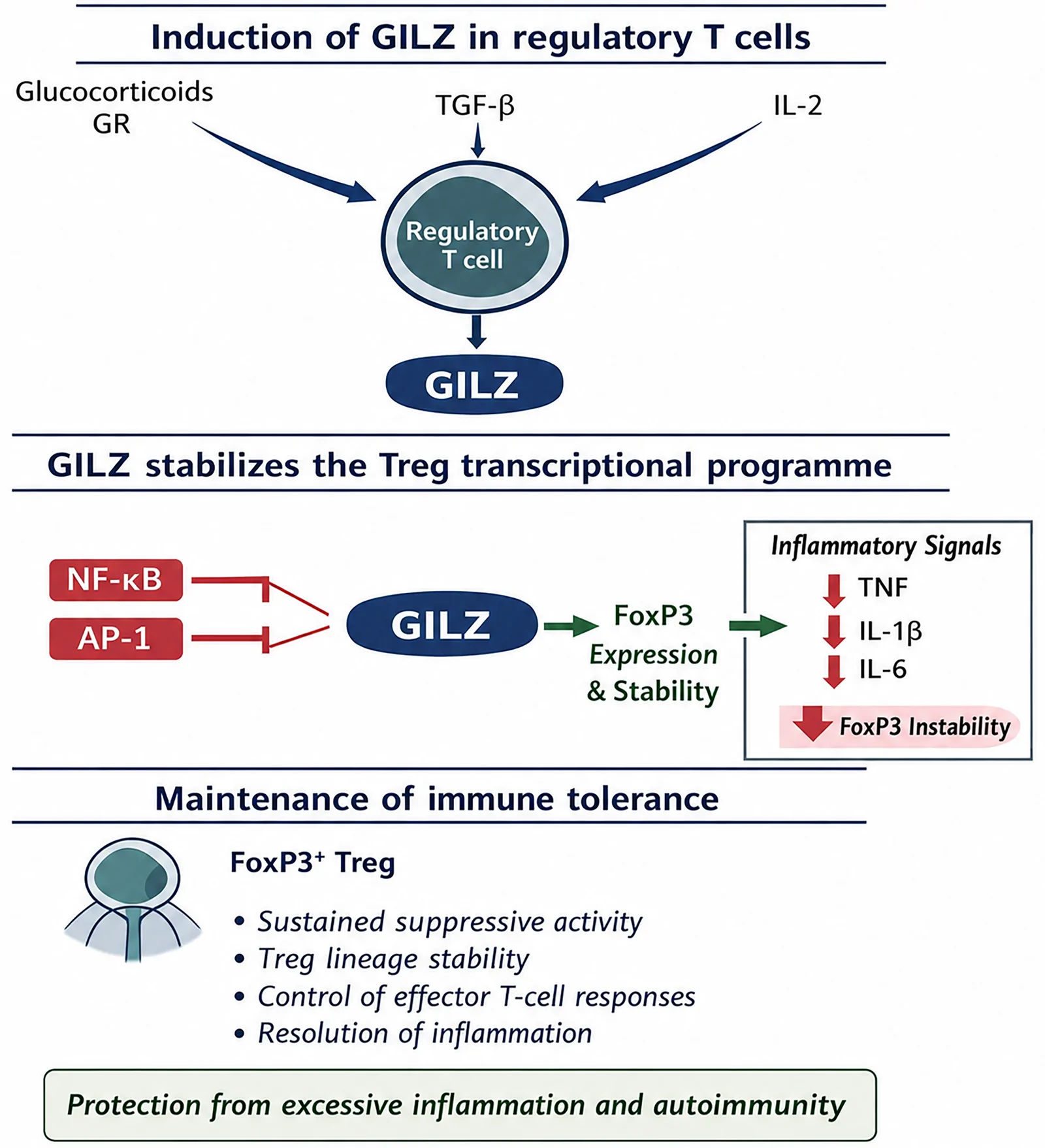
GILZ regulation of regulatory T-cell stability and function. Glucocorticoids, TGF-β, and IL-2 induce GILZ expression in regulatory T cells. GILZ supports the Treg transcriptional program by inhibiting NF-κB- and AP-1-dependent inflammatory signaling and promoting FoxP3 expression and stability. These actions help maintain Treg suppressive function in inflammatory environments containing TNF, IL-1β, and IL-6. Through preservation of Treg lineage stability and regulatory activity, GILZ contributes to peripheral immune tolerance and protection against excessive inflammation and autoimmunity. Created with BioRender.com and Adobe Illustrator.

Mechanistically, GILZ stabilizes the Treg transcriptional program by antagonizing the pro-inflammatory transcription factors NF-κB (2) and AP-1 (20), thereby promoting FoxP3 expression and maintaining lineage stability (**Figure 4**) (23). This is particularly relevant in inflamed tissues, where cytokines such as TNF, IL-1β, and IL-6 can compromise FoxP3-dependent regulatory activity and destabilize Treg identity (72, 73). GILZ helps preserve Treg suppressive function by counteracting these inflammatory signals, thereby supporting immune homeostasis (20, 23, 31). The ability of GCs to reinforce Treg responses is now viewed as mediated, at least in part, through GILZ induction, highlighting a physiological axis linking GC signaling to the preservation of peripheral tolerance and protection from excessive inflammatory and autoimmune responses (**Figure 4**) (21, 23, 36).

### 4.2 CD4+ T helper cells

GILZ is constitutively expressed in naïve CD4+ T cells but is downregulated after TCR engagement in pro-inflammatory environments (2, 23). This reduction is necessary for efficient helper T-cell activation, as sustained GILZ expression inhibits TCR-dependent signaling, limits proliferation, and suppresses effector cytokine production (2, 23). The best-defined role of GILZ in CD4+ T-cell biology is the regulation of the Th17/Treg axis, in which GILZ suppresses RORγt expression and activity, reducing IL-17 production (74), while enhancing TGF-β-dependent FoxP3 induction and Treg differentiation (**Figure 5**) (23, 37). As a result of these complementary actions, GILZ shifts CD4+ responses away from inflammatory Th17 programs toward immunoregulatory Treg phenotypes (46, 74).

**Figure 5:**
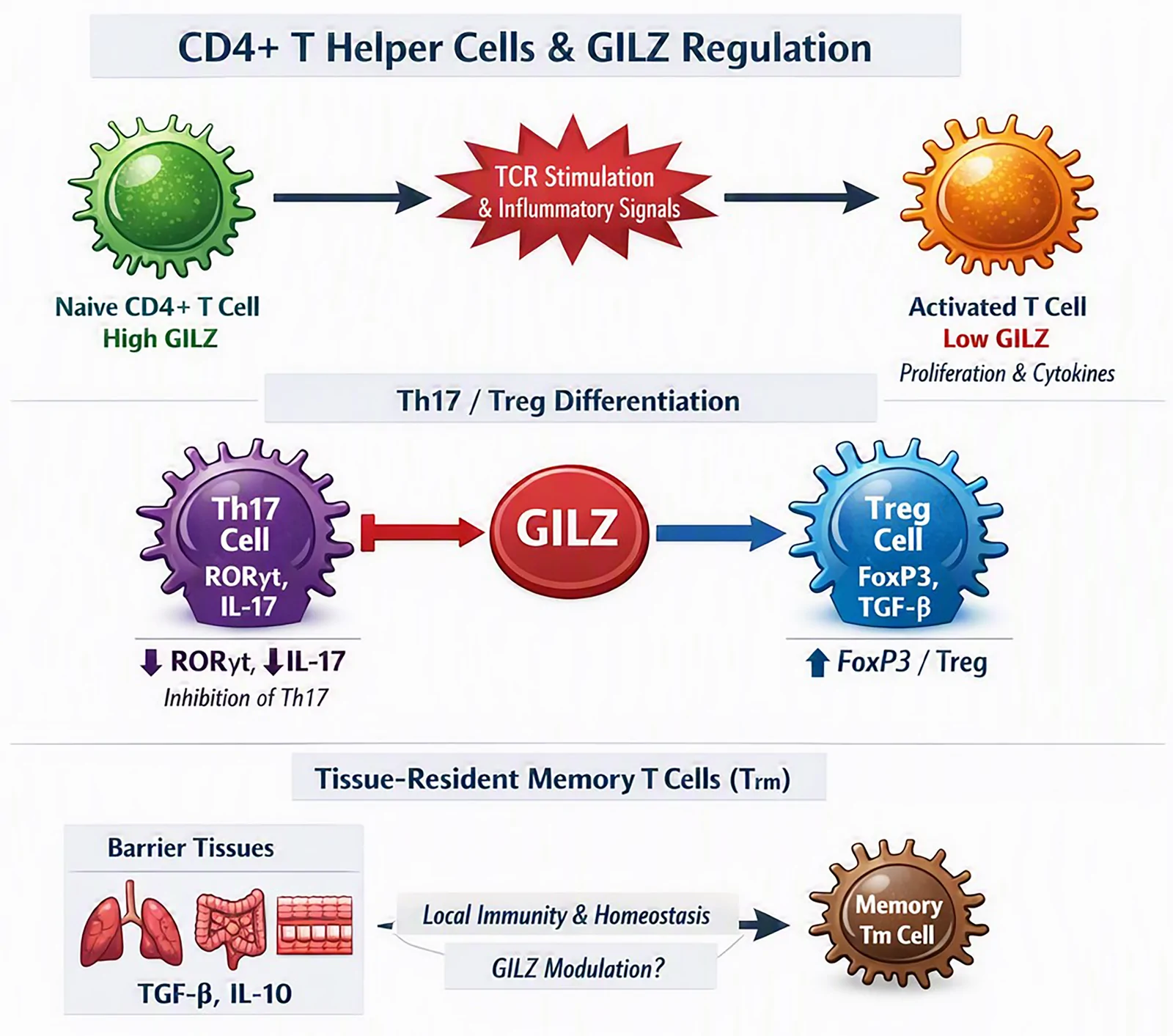
GILZ regulation of CD4^+^ T-cells. GILZ is highly expressed in naïve CD4^+^ T cells and is downregulated following TCR stimulation, allowing efficient T-cell activation. GILZ suppresses Th17-associated RORγt and IL-17 expression during differentiation and promotes TGF-β-dependent FoxP3 induction and Treg development. Also illustrated is a proposed role for GILZ in tissue-resident memory T-cell (T_RM_) biology via interactions with tissue-derived signals, including TGF-β and IL-10. Created with BioRender.com and Adobe Illustrator.

This notion aligns with evidence that GILZ induction attenuates disease severity in experimental colitis, arthritis, and autoimmune neuroinflammation, whereas GILZ deficiency exacerbates Th17-driven pathology (6, 12). In contrast, the influence of GILZ on Th1 differentiation is less consistent across experimental systems (75, 76). Although GILZ can modulate signaling pathways involved in Th1 responses, its effects on T-bet-dependent lineage commitment appear modest compared with its actions on the Th17/Treg axis (6, 23, 37). This selectivity may have therapeutic relevance, as enhancing GILZ activity could preferentially restrain pathogenic Th17 immunity while preserving protective Th1 responses (74).

Tissue-resident memory T (TRM) cells provide another potential context for GILZ activity (7, 77). TRM populations within barrier tissues, including the intestine, lung, and skin, are regulated by local factors such as TGF-β and IL-10 (78) that intersect with GILZ-dependent signaling (77) (**Figure 5**). Although direct evidence remains limited, the established roles of GC signaling and TGF-β responsiveness in TRM differentiation suggest that GILZ may contribute to the establishment or maintenance of tissue residency (79), particularly in chronic inflammatory settings (23, 78). Furthermore, the effects of GILZ on helper T-cell differentiation are not purely T-cell intrinsic, as its expression in dendritic cells and macrophages modifies the cytokine environment linked to antigen presentation and differentiation, thereby influencing (indirectly) CD4+ T-cell fate (28, 58). This indicates that the immunological effects of GILZ reflect both cell-intrinsic and cell-extrinsic regulatory mechanisms (23, 36, 58, 80).

### 4.3 CD8+ T cells

Like CD4+ T cells, naïve CD8+ T cells express GILZ under steady-state conditions; this expression declines rapidly after antigen-specific activation (7, 71). This suggests that transient GILZ downregulation is necessary for efficient CTL activation and effector differentiation, limits responsiveness to antigen stimulation, and restrains engagement of effector transcriptional programs (71, 80). Consistent with this notion, sustained GILZ expression restricts terminal effector differentiation while favoring stem-like and memory precursor phenotypes (71, 81), indicating a role in balancing immediate cytotoxic activity with long-term immune persistence (9, 82).

Exhausted CTLs arise during chronic antigen exposure, particularly in persistent viral infections and tumor microenvironments, and are characterized by a progressive loss of effector functions and distinct transcriptional programs (83, 84). Although a direct role for GILZ in CD8^+^ T-cell exhaustion has not been established, GILZ suppresses T-cell activation and effector differentiation and acts as a checkpoint that regulates CD8^+^ T-cell responsiveness (7, 71). These functions suggest a potential contribution to the development or maintenance of exhaustion-associated states (83). Moreover, because GR signaling participates in exhaustion-associated gene networks in tumor-infiltrating T cells, GILZ may also influence the establishment or persistence of these states (85). Whether GILZ promotes exhaustion or supports T-cell adaptation to chronic stimulation remains unclear.

### 4.4 B cells and humoral immunity

Beyond their role in humoral immunity, B cells contribute to immune regulation and the maintenance of peripheral tolerance through specialized regulatory B-cell (Breg) populations that suppress immune responses primarily by producing IL-10 and, in some subsets, TGF-β (86, 87). GILZ is expressed in B cells and is potently upregulated by GCs, thereby contributing to the regulation of B cell survival, sensitivity to apoptosis, and inflammatory cytokine production (88). Since IL-10, TGF-β, and GCs are established inducers of GILZ, and since IL-10- and TGF-β-driven programs are central to Breg biology, it is plausible that GILZ contributes to Breg stability and suppressive function (23, 58, 87), analogous to its established role in supporting regulatory T-cell differentiation and maintenance.

In addition to its potential role in Bregs, GILZ may influence other aspects of B-cell differentiation. Given the importance of GC signaling in regulating germinal center responses, plasma cell generation, and antibody production (3, 70), GILZ could plausibly modulate pathways involved in plasma cell differentiation or immunoglobulin class-switch recombination, although direct evidence remains limited (86, 87). Whether GILZ fulfils a role in Breg maintenance and function comparable to that seen in Tregs, or regulates the generation and function of antibody-producing B-cell subsets, remains to be determined.

## 5 Resolution of inflammation and immunometabolic regulation

Resolution of inflammation provides a particularly useful biological framework for integrating the apparently diverse functions of GILZ described throughout this review, as it is an active, highly regulated process distinct from the mere cessation of inflammatory stimuli (44, 89). From this perspective, GILZ is viewed not simply an inhibitor of inflammatory initiation, but rather a component of a broader regulatory program that connects inflammatory restraint with macrophage plasticity, efferocytosis, metabolic adaptation, tissue repair, and restoration of homeostasis (59). GILZ expression increases during the resolution phase of acute inflammation, coinciding with macrophage transition toward reparative states, enhanced efferocytosis, and increased production of anti-inflammatory mediators, including IL-10 and TGF-β (8, 41). GILZ also contributes to inflammatory resolution by promoting expression of Annexin A1, a glucocorticoid-induced pro-resolving mediator, suggesting that some glucocorticoid effects are coordinated through a GILZ-Annexin A1 regulatory axis rather than solely through suppression of inflammatory signaling.

Loss of GILZ delays macrophage reprogramming and efferocytosis, resulting in defective resolution of inflammation and impaired restoration of tissue homeostasis (41, 43). This distinguishes GILZ from classical anti-inflammatory mediators, supporting its classification as a pro-resolving rather than purely immunosuppressive molecule (8, 41, 43). Because suppressing inflammation and promoting resolution are distinct therapeutic goals, inhibiting inflammatory initiation alone may not restore homeostasis in chronic inflammation (41, 90). Collectively, these findings support the view that GILZ participates in regulatory networks linking inflammatory resolution, metabolic adaptation, and restoration of immune homeostasis; however, the extent to which GILZ directly orchestrates these interconnected processes remains incompletely resolved (**Figure 6**). In this context, GILZ represents a promising target for restoring immune homeostasis rather than broadly suppressing immune function (31, 41, 43).

**Figure 6:**
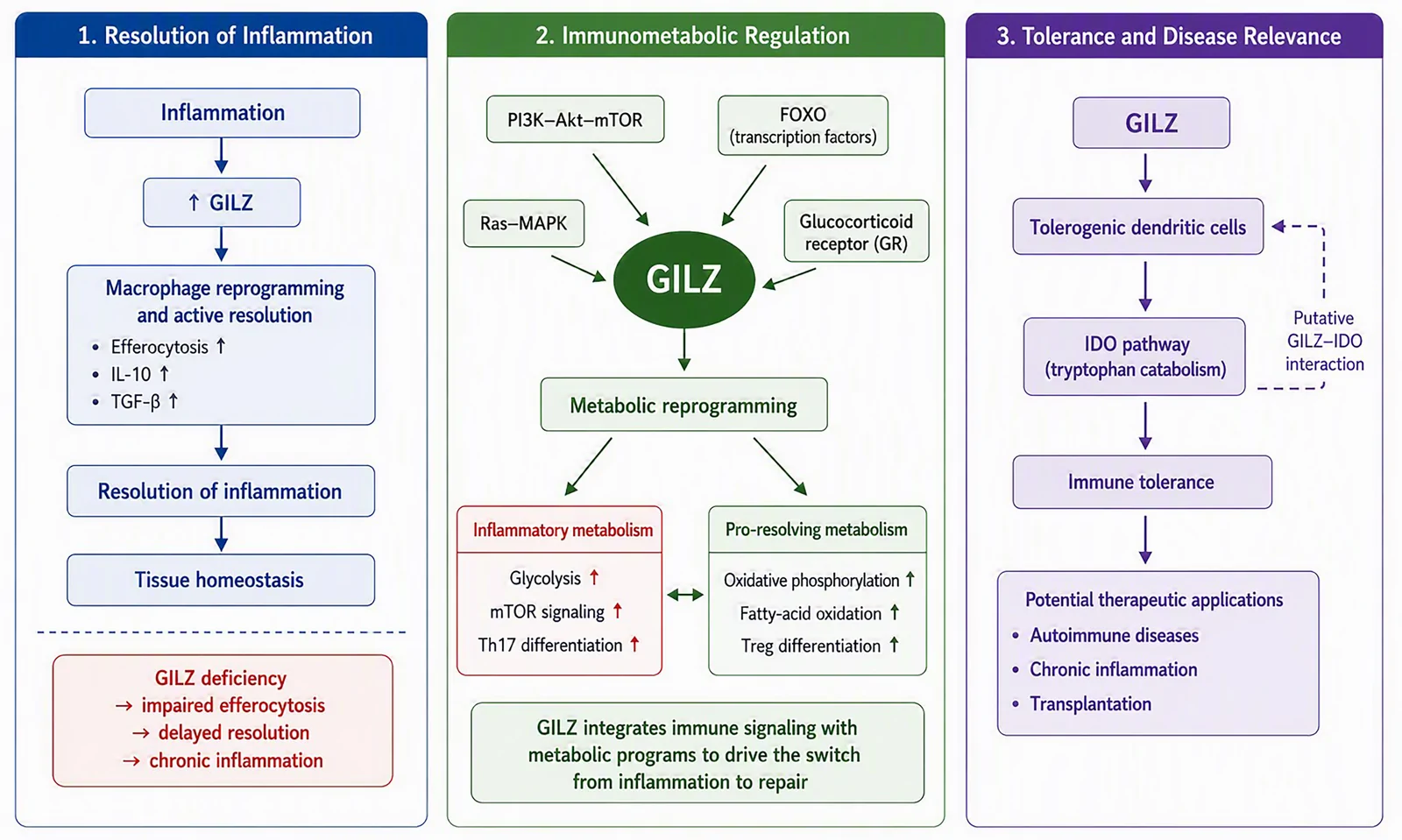
GILZ associations with inflammatory resolution and immunometabolic regulation. During inflammation resolution, increased GILZ promotes macrophage reprogramming, efferocytosis, and production of IL-10 and TGF-β, and expression of Annexin A1, restoring tissue homeostasis, whereas GILZ deficiency is associated with defective resolution and persistent inflammation. GILZ also has been linked to immune signaling and metabolic pathways, favoring a shift from inflammatory metabolism and Th17 differentiation toward pro-resolving metabolism and Treg development. In addition, GILZ supports tolerogenic dendritic-cell function and may interact with IDO-dependent pathways that contribute to immune tolerance.

Immunometabolic studies show that cellular metabolic programs actively shape immune cell phenotype and function (91) and that the transition from inflammatory to reparative states is inseparable from metabolic reprogramming (92). Several GILZ-regulated signaling pathways, including PI3K-Akt-mTOR, FOXO transcription factors, Ras-MAPK, and the GR, are closely linked to metabolic regulation, placing GILZ at the interface of immune signaling and cellular metabolism (17, 51). In macrophages, GILZ expression is associated with transcriptional and functional changes involving mitochondrial pathways and an anti-inflammatory macrophage phenotype (8, 92, 93), although available studies have not yet established whether these metabolic changes are direct consequences of GILZ activity.

GILZ promotes a tolerogenic dendritic-cell phenotype that limits antigen-specific T-cell activation, while IDO-expressing dendritic cells modulate local tryptophan catabolism and generate tolerogenic signals that shape adaptive immune responses (61, 94). Although a direct mechanistic link between GILZ and IDO has not been fully defined, available evidence suggests that GILZ-mediated immune regulation extends beyond transcriptional control of inflammatory pathways to include metabolic mechanisms that shape the local immune microenvironment (58, 61). This interaction is relevant in transplantation and autoimmune disease, where GC-induced or tolerogenic dendritic cell--mediated immune tolerance has been associated with IDO-dependent mechanisms (61, 95).

Metabolic programming also shapes T-cell fate decisions that may intersect with GILZ-dependent immune regulation. Increased glycolytic flux and mTOR signaling support the differentiation of pro-inflammatory Th17 cells (74, 96), whereas AMPK activation, oxidative metabolism, and fatty-acid oxidation favor the development and maintenance of Tregs (23, 97). Because GILZ modulates PI3K-Akt-mTOR activity and integrates signals from the glucocorticoid and TGF-β pathways, its regulatory effects on the Th17/Treg axis may involve metabolic reprogramming alongside direct transcriptional control (7, 23, 68). This mechanistic dimension remains to be fully defined but could have important implications for therapeutic targeting of GILZ in autoimmune and inflammatory diseases characterized by Th17-driven pathology (7, 23).

## 6 GILZ in tissue homeostasis beyond the immune system

GILZ expression in non-immune tissues indicates biological significance that extends well beyond classical immune regulation. The best-characterized non-immune roles are in the intestinal epithelium and liver, with evidence from experimental models and human tissue studies (98–104). A summary of GILZ activity across additional tissue compartments is provided in **Table 1**.

**Table 1:** GILZ expression and functional roles in selected non-immune tissue compartments.

Tissue	GILZ expression context	Key functional role	Disease/therapeutic relevance ^1^	Type of study ^2^	Reference
Endothelial cells	Constitutive; induced by GCs	Regulates ICAM-1, VCAM-1, and E-selectin; limits leukocyte recruitment to sites of tissue injury	Vascular inflammation; resolution of tissue injury	*In vitro* human endothelial-cell studies; animal models of vascular inflammation	(37)
Adipose tissue	GC-regulated; present in adipocytes and adipose immune cells	Participates in adipogenesis, lipid metabolism, and the adipose immune microenvironment	GC-induced metabolic complications; obesity-associated immune dysregulation	*In vitro* adipocyte studies; animal/tissue studies; mechanistic review evidence	(10, 105)
Bone (osteoblasts)	Constitutive; GC-regulated in osteoblast precursors	Regulates osteoblast differentiation and the bone formation–resorption balance; loss impairs osteogenic differentiation	Glucocorticoid-induced osteoporosis	*In vitro* osteoblast/mesenchymal-cell studies; animal/preclinical evidence	(21, 105, 106)
Nervous system	Expressed in neurons and glial cells	Neuroimmune regulation; potential mediation of GC effects on neuroinflammation via bidirectional neuro–immune feedback circuits	Multiple sclerosis; traumatic brain injury; stress-induced immune modulation	Animal models; neural/tissue-expression studies; limited human observational evidence	(34, 105)
Cardiovascular tissue	Expressed in immune and cardiovascular-associated cells and acts as a GC-responsive regulator of inflammation.	Promotes immune tolerance and suppresses excessive inflammatory signaling, contributing to the regulation of leukocyte recruitment, cytokine production, and cardiovascular immune homeostasis.	Atherosclerosis, myocarditis, and vasculitis; a potential therapeutic target for controlling cardiovascular inflammation.	*In vitro* vascular-cell studies; animal models; translational/clinical-context evidence	(107)

GC, glucocorticoid; ICAM-1, intercellular adhesion molecule-1; VCAM-1, vascular cell adhesion molecule-1.

^1^Labeled *potential* when no direct clinical evidence has established a causal role for GILZ.

^2^Indicates the main evidence source for each summarized GILZ finding.

### 6.1 Intestinal epithelium

In intestinal epithelial cells, GILZ helps maintain barrier integrity and regulate local inflammatory responses (98). Reduced epithelial GILZ expression is linked to impaired barrier function, heightened inflammatory signaling, and defective mucosal reconstitution after injury (98, 99). Evidence from human tissue sections demonstrates that decreased GILZ expression is accompanied by intestinal epithelial inflammation, underscoring the anti-inflammatory role of GILZ in the gut (100). Emerging evidence further suggests that GILZ interacts with pathways involved in epithelial sensing of luminal microbial and inflammatory cues, including mucin-associated and TLR-dependent signaling networks (12, 101); however, direct mechanistic links remain incompletely characterized. Because disruption of epithelial barrier function and loss of immune tolerance to commensal microbiota are central and interrelated features of inflammatory bowel disease, dysregulated GILZ expression may contribute to disease pathogenesis and the maintenance of mucosal inflammation (98, 101).

### 6.2 Hepatic biology

In the liver, GILZ is expressed in hepatocytes and hepatic stellate cells, where it has been implicated in GC-mediated regulation of hepatic glucose metabolism, lipid homeostasis, and inflammatory responses (102, 103). Hepatic GILZ has been implicated in GC-regulated gluconeogenesis, attenuation of lipopolysaccharide-induced inflammatory responses in hepatocytes (104), and modulation of hepatic stellate cell activation (102), a central process in hepatic fibrosis development (102, 104). The liver occupies a unique immunological position as a site of continuous exposure to antigens and microbial products arriving through the portal circulation. Given the central role of Kupffer cells in maintaining tolerance to gut-derived antigens and microbial products, and the emerging evidence that GILZ restrains hepatic inflammatory responses, GILZ expression in hepatic immune and parenchymal cells may contribute to the liver’s tolerogenic immune environment, although direct evidence demonstrating a causal role in maintaining hepatic immune tolerance remains limited (102, 104). Dysregulation of hepatic GILZ expression has been proposed to contribute to chronic liver inflammation and fibrogenesis, while its potential role in metabolic liver diseases remains an area of active investigation.

### 6.3 Additional tissue compartments

Evidence for GILZ expression and function has been reported in endothelial cells, adipose tissue, bone, the nervous system, and cardiovascular-associated tissues [107]. However, evidence strength varies across compartments and derives from diverse sources, including *in vitro* studies, animal models, human tissues, and limited clinical observations. **Table 1** summarizes tissue-specific findings, identifies the supporting study type and references, and distinguishes experimentally demonstrated functions from largely preclinical or inferential associations. These data highlight the broad biological activity of GILZ while underscoring the need for tissue-specific validation and assessment of potential off-target effects before systemic GILZ-directed therapies are pursued.

## 7 Clinical implications and therapeutic opportunities

### 7.1 Autoimmunity and transplantation

The capacity of GILZ to stabilize Treg identity, attenuate Th17-driven pathology, and promote tolerogenic DC programming makes it an attractive candidate for autoimmune diseases in which peripheral tolerance failure is central to pathogenesis (7, 31). In multiple experimental models of autoimmune and chronic inflammatory disease, including lupus, arthritis, and inflammatory bowel disease, increased GILZ expression or activity has been associated with reduced disease severity and improved immune regulation (1, 14). In lupus and inflammatory bowel disease (108), where the Th17/Treg imbalance is directly implicated in pathology, enhancing GILZ may therefore help restrain pathogenic effector responses while supporting regulatory immune pathways (74, 109). Similarly, in rheumatoid arthritis, where Th17-associated inflammatory pathways contribute to joint pathology, GILZ-based approaches have been proposed as a complement to existing cytokine-targeted therapies by promoting regulatory immune mechanisms rather than directly blocking inflammatory mediators (109).

Given GILZ’s established role in promoting tolerogenic dendritic-cell function, GILZ-based approaches to generating tolerogenic DCs ex vivo have been proposed as potential adjuncts to conventional immunosuppressive strategies in transplantation (55, 110). Ex vivo generated tolerogenic DCs expressing elevated levels of GILZ show reduced immunogenicity and enhanced regulatory properties in experimental systems, offering a pathway to tolerance induction with reduced systemic drug burden (16, 54). Because GILZ promotes Treg generation and FoxP3 induction, augmenting GILZ has been proposed as a strategy to support the stability and function of Treg-based cellular therapies under inflammatory conditions (23), where preserving regulatory lineage commitment remains a significant challenge.

### 7.2 Cancer immunotherapy

The role of GILZ within the tumor microenvironment may be context-dependent, creating both challenges and opportunities for immunotherapeutic intervention (9). As a downstream mediator of GR signaling, GILZ may contribute to immunoregulatory programs that limit anti-tumor immunity, although its specific role in tumor-associated CD8^+^ T-cell dysfunction remains incompletely defined (85). Preclinical studies indicate that inhibiting glucocorticoid receptor signaling in tumor-infiltrating lymphocytes can enhance anti-tumor immunity, raising the possibility that downstream mediators such as GILZ may represent therapeutically relevant targets (85). Conversely, in tumor settings where chronic inflammation promotes disease progression, enhancing GILZ activity could theoretically help regulate pathological inflammatory responses (24, 54). The net therapeutic effect of GILZ modulation is therefore tumor-type-specific and requires evaluation against the immunological microenvironment of each indication (54, 85).

### 7.3 Sepsis and inflammatory resolution disorders

Sepsis is marked by a dysregulated host response that often progresses from an initial hyperinflammatory state to a subsequent immunosuppressive phase, reflecting impaired regulation of inflammatory resolution. Given its established roles in anti-inflammatory signaling, inflammatory resolution, and macrophage functional reprogramming, GILZ may be relevant to both phases of the septic response (111). During the hyperinflammatory phase, stress-hormone-induced endogenous GILZ may limit excessive amplification (112). Conversely, persistent or excessive GILZ expression during the immunosuppressive phase could contribute to impaired host defense (113, 114), although the precise role of GILZ in sepsis-associated immunoparalysis remains incompletely understood. Dynamic rather than constitutive modulation of GILZ will therefore be necessary in this context.

### 7.4 Targeted GILZ modulation beyond systemic glucocorticoids

The most significant therapeutic implication of GILZ biology is recapitulating selected GC immunoregulatory effects while avoiding many of the metabolic, endocrine, and skeletal toxicities associated with chronic systemic GC therapy (1, 25). Several strategies have been proposed to achieve this goal, including cell-targeted delivery of GILZ-based therapeutics, pharmacological approaches to enhance or sustain GILZ activity independently of systemic GC exposure, and cell-based therapies using GILZ-enriched tolerogenic dendritic cells or Treg cells (16, 23, 25). A complementary approach is developing selective GR modulators that preferentially induce beneficial downstream mediators such as GILZ while reducing unwanted systemic effects (7, 106). Although each strategy faces substantial challenges related to target specificity, delivery, and durability of response, together they show how mechanistic insights into GILZ biology are informing the development of next-generation immunomodulatory therapies (7, 25).

Beyond its potential as a therapeutic target, GILZ may also serve as a useful pharmacodynamic biomarker of GR activity (7). Current clinical assessment of chronic GC exposure relies primarily on monitoring endocrine suppression and treatment-related toxicities, including HPA-axis suppression, adrenal insufficiency, hyperglycemia, dyslipidemia, and GC-induced osteoporosis, rather than direct measures of immunological GC responsiveness (115, 116). GILZ provides a biologically proximal readout of GC signaling because it is rapidly induced downstream of GR activation in leukocytes and can be quantified in circulating immune cells (13, 82, 112). Studies in rheumatoid arthritis have shown that peripheral blood GILZ expression varies with GC exposure and disease activity, supporting its potential utility as a biomarker of GC responsiveness and treatment effects (105, 117). Because GILZ expression is closely linked to GC signaling and can be quantified in circulating immune cells, future development and validation of standardized GILZ-based assays may facilitate individualized GC therapy, improve monitoring of pharmacodynamic responses, and help identify patients with altered GC responsiveness who may benefit from alternative immunomodulatory strategies (105, 112, 117).

## 8 Conclusions and future directions

A central challenge in the therapeutic exploitation of GILZ is its profound context dependence. The biological effects of GILZ vary with cellular identity, activation state, and tissue microenvironment, making uniform outcomes across immune and non-immune compartments unlikely (118). Although L-GILZ has established physiological functions *in vivo*, evidence supporting endogenous expression and specialized biological roles for several other reported GILZ isoforms remains limited. Consequently, systemic GILZ modulation may produce unpredictable or unintended effects in tissues beyond the intended target. Successful translation of GILZ-based therapies will require precise cell- and tissue-specific targeting that accounts for context dependence while minimizing off-target effects. Because evidence for isoform-specific expression and function remains limited, integrated transcriptomic, proteomic, spatial, and genetic approaches are needed to establish true *in vivo* specialization and guide physiologically informed, isoform-targeted immune modulation. Understanding GILZ as a context-dependent regulator provides a conceptual framework for integrating its diverse biological functions across immune and non-immune systems.
